# Decreased risk of falls in patients attending music sessions on an acute geriatric ward: results from a retrospective cohort study

**DOI:** 10.1186/s12906-019-2484-x

**Published:** 2019-03-28

**Authors:** Julia Chabot, Olivier Beauchet, Shek Fung, Isabelle Peretz

**Affiliations:** 1 Department of Medicine, Division of Geriatric Medicine, Sir Mortimer B. Davis - Jewish General Hospital and Lady Davis Institute for Medical Research, McGill University, 3755 chemin de la Côte-Sainte-Catherine, Montréal, QC, H3T 1E2 Canada; 2 Department of Medicine, Division of Geriatric Medicine, St. Mary’s Hospital Center, McGill University, 3830 Lacombe Avenue, Montreal, QC, H3T 1M5 Canada; 3 International Laboratory for Brain, Music and Sound Research (BRAMS), Montreal, Quebec, H3C 3J7 Canada; 4 Dr. Joseph Kaufmann Chair in Geriatric Medicine, Faculty of Medicine, McGill University, Montreal, Quebec, Canada; 5 Centre of Excellence on Longevity of McGill Integrated University Health Network, Quebec, Canada; 6 Department of Psychology, University of Montreal, Montreal, Quebec, H3C 3J7 Canada

**Keywords:** Music, Fall, Elderly

## Abstract

**Background:**

Music has been shown to improve health and quality of life. It was suggested that music may also have an impact on gait stability and fall risk. Yet, few studies have exploited music in the hospital setting, and even less so in the geriatric population. Our objective was to examine the influence of music listening on the risk of falls by comparing the Morse Fall Scale score in patients admitted to a Geriatric Assessment Unit (GAU) who attended music listening sessions and in patients who did not attend music sessions.

**Methods:**

This was a retrospective cohort study (mean follow-up 13.3 ± 6.8 days) which took place in a GAU, St. Mary’s Hospital Center, Montreal. A total of 152 charts of participants, with a mean age of 85.7 ± 6.4 years and 88.2% female were reviewed and included. There were 61 participants exposed to the music listening sessions group and 91 in the non-exposed group matched for age, sex, cause and season of admission, and living situation. One-hour music sessions were provided to the patients by volunteer musicians. The Morse Fall Scale score upon admission and discharge as well as its variation (change from before to after exposure) were used as outcomes. Age, sex, living situation, reason for admission, season of admission, Mini Mental Status Examination score, number of therapeutic classes taken daily upon admission, use of psychoactive drugs upon admission and length of stay were used as covariates.

**Results:**

The Morse Fall Scale score decreased significantly in the exposed group compared to the non-exposed group (*p* = 0.025) and represented a small to medium-sized effect, *d* = 0.395. The multiple linear regression model showed a significant association between the decrease of the Morse Fall Scale score and music exposure (B = − 17.1, *p* = 0.043).

**Conclusion:**

Participating in music listening sessions was associated with a decreased risk of falls in patients admitted to a GAU. Further controlled research is necessary to confirm these findings and to determine the mechanisms by which music listening impacts fall risk.

**Trial registration:**

Clinical trial registry: ClinicalTrials.gov. Registration number: NCT03348657 (November 17th, 2017). Retrospectively registered.

## Background

Music is often used as a non-verbal means of emotional expression [1]. As communication can be impaired in the elderly due to cognitive impairment and diseases, music can be used to recreate communication between the patients and their environment [2, 3]. Music therapy has long been used to improve communication, health and quality of life. Music is also known to regulate pain, mood and anxiety levels [4–10]. In the geriatric population, music listening has been shown to decrease depressive symptoms [11–15] and neuropsychiatric symptoms such as agitation and anxiety [9, 16]. As a result, the use of music is recommended by national guidelines to control the behavioural symptoms of patients in long-term care facilities [17]. Despite the demonstrated positive benefits of music for health and behavioural outcomes, very few studies using music have been performed in the hospital environment and even fewer on short-stay geriatric units.

Older adults are the fastest-growing group of patients admitted to hospital, and the age-related burden of non-fatal health outcomes is one of the main challenges faced by hospitals. Thus, assessing and addressing the needs of the growing number of geriatric patients is necessary [18]. One of those age-related burdens is related to falls [19–21]. Falls are highly frequent in geriatric patients, particularly on short-stay geriatric units, with a prevalence of up to 30% [20]. Falls are associated with increased length of hospital stay, high health-care costs and negative non-fatal health outcomes including multi-morbidities and related disabilities [19–23].

Previous research has shown that music may decrease the risk of falls [24, 25]. For example, it was shown that the rhythm of music, combined with physical exercise, can improve measures of gait stability [24, 26]. In older community dwellers, music-based programs have demonstrated that improvement of gait stability decreased the risk of falls [24, 25]. We therefore hypothesized that music listening may decrease the risk of falls of geriatric patients admitted to a short stay unit.

This study aimed to examine the influence of music listening on the risk of falls in patients admitted to a Geriatric Assessment Unit (GAU) by comparing the Morse Fall Scale (MFS) score in patients who attended music listening sessions and in patients who did not attend these music sessions.

## Methods

### Participants

The participants were a convenience sample of inpatients who were admitted to the GAU of St. Mary’s Hospital Center (Montreal, Quebec, Canada) between October 2014 and May 2016. The selection criteria were a first admission to the GAU and a length of stay between 5 and 31 days. A total of 571 inpatients were admitted during the period of recruitment. Data was obtained retrospective chart review. As participation to the music listening sessions was voluntary, the patients from the exposed group to the music sessions were matched to patients who did not attend the music sessions (non-exposed group) according to age (+/− 3 years), sex, season of admission, cause of admission, and living situation (at home or institutionalized).

In total, 152 (26.6%) patients (mean age 85.7 ± 6.4 years, 88.2% female) met the selection criteria and were included for analysis in this retrospective cohort study. Sixty-one patients were included in the exposed group (music sessions) and 91 patients were included in the non-exposed group.

### Music listening sessions

The music listening sessions took place in the family room of the GAU. Volunteer musicians provided an average of four one-hour music listening sessions per week. Most of these volunteers/musicians were McGill University students playing the piano, guitar, saxophone, and violin, or singing. On average, four to six patients attended these mini-concerts on a voluntary basis. The exposed group participated in average to 1.5 music listening sessions (minimum 1, maximum 4) which lasted 1 h each.

### Assessment

The charts of the patients who were admitted to the GAU were reviewed using a systematic and standardized procedure for collection of data. Collected information included age, gender, reason for admission (mobility disorders, neuropsychiatric disorders and organ failure), living situation prior to admission, season of admission (Montreal being a Nordic city with significant weather changes), Mini Mental Status Examination score [27] at the time of admission, number of therapeutic medication classes taken daily (excluding laxatives) upon admission and discharge, use of psychoactive drugs (i.e., benzodiazepines, antidepressants or neuroleptics) upon admission and discharge, MFS score upon admission and discharge, and length of stay.

### Risk of falls

The risk of falls was measured using the Morse Fall Scale (MFS). This is a rapid and simple method to assess the probability that a patient will fall. The total score is out of 125 and includes 6 items: history of previous falls, presence of a secondary diagnosis (i.e. more than one medical diagnosis in the patient’s chart), use of an ambulatory aid (none, cane, walker), presence of intravenous therapy, gait and transfers (normal, weak, impaired) and the patient’s mental status (oriented towards own ability or not). The score is further divided into 3 risk levels: low risk (< 25 points), medium risk (25–44 points) and high risk (≥ 45 points) [23, 28]. If a patient is classified in the medium risk category, then universal fall prevention interventions are applied (environmental modifications, and consideration regarding ambulation). However, if a patient is classified in the high risk category, then individualized fall interventions are applied in addition to universal fall prevention interventions (padded furniture, moving the patient near the nursing station, consider use of a transfer belt, etc.). At St. Mary’s Hospital Center, the MFS is completed within 24 h of admission, upon transfer to a different unit, within 48 h following a fall, and every week. For this study, the first MFS score (upon admission) and the last MFS score (prior to discharge) were collected.

### Standard protocol approvals, registrations, and participant consents

The study was conducted in accordance with the ethical standards set forth in the Helsinki Declaration (1983). The study was approved by the Research Ethics Committee of St. Mary’s Hospital Center (SMHC #14–31). As this was a retrospective study, written or verbal consent from the participants was not obtained. This study was retrospectively registered: Clinical trial registry: ClinicalTrials.gov. Registered number: NCT03348657 (November 17th, 2017).

### Statistical analysis

Participants were separated into 2 groups (exposed versus non-exposed) based on whether they participated in the music listening sessions. The participants’ characteristics were summarized using means and standard deviations or frequencies and percentages, as appropriate (see Table 1). For the analysis, change in the MFS score was calculated as follows: ((discharge - upon admission)/ ((discharge + upon admission)/2)) × 100 [29]. Comparisons between groups were based on unpaired *t*-tests and Chi-square tests, as appropriate. Multiple linear regression was performed to examine the association between changes in MFS score (dependent variable) and participation in the music listening sessions (independent variable) adjusted for participant baseline characteristics which are well recognized risk factors for falls in the literature [22]. These risk factors were gender, living situation, cognitive performance, number of therapeutic classes daily taken and use of psychoactive drugs. We did not adjust on other participant characteristics because they are not risk factor for falls and there was no significant difference between groups. *P*-values less than 0.05 were considered statistically significant. All statistical analyses were performed using SPSS (version 24.0; SPSS, Inc., Chicago, IL).

**Table 1:** Baseline characteristics of participants who were exposed and non-exposed to the music listening sessions (*n* = 152)

	Total population (*n* = 152)	Music listening sessions		*P*-Value^a^	Effect size (Cohen’s d)
		Exposed group (*n* = 61)	Non-exposed group (*n* = 91)		
Age, mean ± SD (years)	85.7 ± 6.4	85.7 ± 6.6	85.8 ± 6.3	0.915	0.018
Female, n (%)	134 (88.2)	53 (86.9)	81 (89.0)	0.691	
Living situation home^b^, n (%)	111 (73.0)	45 (73.8)	66 (72.5)	0.866	
Reason for admission, n (%)					
Mobility disorders^c^	125 (82.2)	47 (77.0)	78 (85.7)	0.376	
Neuropsychiatric disorders^d^	18 (11.8)	9 (14.8)	9 (9.9)		
Organ failure^e^	9 (5.9)	5 (8.2)	4 (4.4)		
Season of admission, n (%)					
Winter	54 (35.5)	22 (36.1)	32 (35.2)	0.999	
Spring	18 (11.8)	7 (11.5)	11 (12.1)		
Summer	20 (13.2)	8 (13.1)	12 (13.2)		
Autumn	60 (39.5)	24 (39.3)	36 (39.6)		
MMSE score, mean ± SD (/30)	21.5 ± 5.6	21.5 ± 6.0	21.6 ± 5.4	0.951	0.011
Therapeutic classes daily taken					
Mean ± SD upon admission	8.2 ± 4.2	8.6 ± 4.3	7.9 ± 4.1	0.270	0.187
Mean ± SD discharge	9.2 ± 4.2	9.7 ± 4.5	9.0 ± 4.0	0.326	0.172
Variation^f^ (%)	14.7 ± 37.9	11.2 ± 24.4	17.1 ± 44.8	0.350	0.015
Use of psychoactive drugs^g^, n (%)					
Upon admission	64 (42.1)	30 (49.2)	34 (37.4)	0.148	0.213
Discharge	61 (40.1)	29 (47.5)	32 (35.2)	0.127	0.177
Length of stay^h^, mean ± SD (days)	13.3 ± 6.8	14.8 ± 7.5	12.2 ± 6.1	**0.019**	0.200

*SD* standard deviation

*MMSE* Mini Mental Status examination

^a^Comparison between exposed group and non-exposed group based on unpaired t-test or Chi-square test, as appropriate; significant *p* values (i.e.; ≤0.05) in bold

^b^Versus institution

^c^Defined as gait and/or balance disorders and/or falls (unintentionally coming to rest on the ground, floor, or other lower level)

^d^Defined as neurocognitive disorder (dementia), delirium or behavioural disorders

^e^Defined as congestive heart failure, chronic lung disease, chronic kidney disease or cirrhosis

^f^Based on the formula: ((discharge - upon admission) / ((discharge + upon admission)/2)) × 100

^g^Antidepressants, benzodiazepines or neuroleptics

^h^Number of days between the first day of admission to the geriatric ward and the last day of hospitalization on the same geriatric ward, determined from the administrative registry of St. Mary’s Hospital Center

## Results

Table 1 shows patient characteristics and MFS scores for exposed and non-exposed groups to music listening sessions. The exposed group had a significantly longer length of stay at the hospital (*p* = 0.019). Table 2 shows the Morse Fall Scale means and risk stratification. More participants from the exposed group (83.6%) were classified as high risk for falls upon admission than from the non-exposed group (69.2%) (*p* = 0.045). The exposed group appeared to have higher MFS scores on admission compared to non-exposed group, but this difference did not reach significance (*p* = 0.078). A significantly greater reduction in the MFS score from admission to discharge in the exposed group when compared to the non-exposed group (*p* = 0.025) was shown. There was a trend to have fewer patients in the exposed group who were classified in the high-risk category upon discharge compared to those in non-exposed group (45.9% versus 52.7 with *P* = 0.408) (Table 2).

**Table 2:** Morse Fall Scale means and risk stratification of participants who were exposed and non-exposed to the music listening sessions at admission and discharge (*n* = 152)

	Total population (*n* = 152)	Music listening sessions		*P*-Value*	Effect size (Cohen’s d)
		Exposed group (*n* = 61)	Non-exposed group (*n* = 91)		
Morse Fall Scale, mean ± SD (/125)					
Mean ± SD upon admission	63.7 ± 23.9	67.9 ± 22.1	60.9 ± 24.7	0.078	0.281
Mean ± SD discharge	45.4 ± 17.3	44.4 ± 18.9	46.0 ± 16.3	0.584	0.097
Variation (%)	−30.7 ± 50.1	− 41.8 ± 47.1	− 23.3 ± 51.0	**0.025**	0.395
MFS estimated risk, n (%)					
Admission					
Low risk	9 (5.9)	3 (4.9)	6 (6.6)	0.668	
Medium risk	29 (19.1)	7 (11.5)	22 (24.2)	0.051	
High risk	114 (75.0)	51 (83.6)	63 (69.2)	**0.045**	
Discharge					
Low risk	13 (8.6)	8 (13.1)	5 (5.5)	0.100	
Medium risk	63 (41.4)	25 (41.0)	38 (41.8)	0.924	
High risk	76 (50.0)	28 (45.9)	48 (52.7)	0.408	

*Comparison between exposed group and non-exposed group Chi-square test

significant *p* values (i.e.; ≤ 0.05) in bold

A multiple linear regression was performed to examine the relationships between variation of the MFS score, group assignment, and patient characteristics (Table 3). The overall regression model was not significant F (8,143) = 1.703, *p* = 0.102, R^2^ = 0.087. However, after adjusting for multiple participants characteristics, a significant decrease in MFS score after the music listening sessions was found (B = − 17.1 and β = − 0.186 with *p* = 0.043).

**Table 3:** Multiple linear regression showing the association between variation^a^ of Morse Morse Fall Scale score and participation to music listening sessions adjusted on participants’ characteristics (*n* = 152)

	Fully adjusted model		
	B	[95% CI]	*P*-value
Music listening session	**−** **17.1**	[−33.6;-0.5]	**0.043**
Length of stay	0.5	[−0.7;1.7]	0.411
Female	15.9	[−9.0;40.8]	0.209
Living situation (home)	−10.2	[−29.5;9.0]	0.294
MMSE score (admission)	−0.6	[−2.1;0.9]	0.446
Therapeutic classes taken daily (admission)	−1.4	[−3.4;0.7]	0.191
Use of psychoactive drugs (admission)	11.1	[−6.4;28.6]	0.211

^a^Variation of Morse Fall Scale score between upon admission and discharge and based on the formula: ((discharge - upon admission) / ((discharge + upon admission)/2)) × 100

β and *p*-value significant (i.e.; ≤0.05) in bold

β: coefficient of regression beta corresponding to a change of Morse Fall Scale score at discharge or of variation of Morse Fall Scale score between upon admission and discharge

*MMSE* Mini Mental Status examination

*CI* confidence interval

## Discussion

There was a significant reduction in the MFS score in the exposed group to music listening sessions compared to the non-exposed group, indicating a decreased risk of falls. Risk factors for falls in the geriatric population are multifactorial and very complex. One of those risk factors is depression. A recent meta-analysis shows that the association between falls and depression in older people has an odds ratio of 1.63 (95% CI: 1.36–1.94) [30]. Considering that music has been associated with an improvement in depressive symptoms in the geriatric population [8, 14, 31], it is possible that the musical exposure indirectly decreased the risk of falls in the studied participants by alleviating depressive symptoms. Our study, however, did not include any indicators of emotional well-being or mood. In the future, questionnaires should be added to this effect.

The reduction of risk of falls in patients attending music session may also be related to other factors. The patients who participated to the music listening sessions may have been more motivated to walk. Since physical activity decreases the risk of falls, the simple walk from bed to the music room may have on its own been sufficient to decrease the risk of falls in participants [32].

Also, music has been previously used as support of physical exercises for gait training in community dweller individuals, patients with Parkinson's disease and older adults living in nursing homes [24, 25, 33]. Considering that falls usually occur while walking in older adults [34] and that gait irregularity has been identified as a marker of gait instability [26], the rhythmic component of music has been used to regularize gait with a synergistic effect combined with physical exercises [26]. Therefore, given the role of music, and particularly the rhythmic aspects of music in gait stability, it is certainly possible that music listening influenced fall risk. Also, in neurosciences, it has been demonstrated that performing, observing or imagining a movement activates the same neural brain network [35]. The equivalence between performed and imagined or observed movement has been used in rehabilitation to improve the physical performance of sportive individuals as well as patients with mobility impairments [36, 37]. Because the rhythmic component of music has been identified as the key element of improvement of gait stability in intervention combining music and physical activity, and because of motor brain equivalences, it could be suggested that listening to music could improve stability of gait and thus reduce the risk of fall in geriatric patients.

The patients of the exposed group to music listening sessions had a significantly longer length of stay compared to the patients from the non-exposed group. Considering that comorbidities and poor functional is associated with a longer length of stay [38], it is possible that the patients from the exposed group had more complex medical issues and were more encouraged to attend the live music sessions because the medical team believed in the potential benefits of exposure to music. To reduce the effect of such confounding variables, we considered the patients’ baseline characteristics in the statistical analyses. After controlling for such effects, the reduction in risk of falls seen in the music listening sessions group remained significant.

The main limitation of our study is the fact it may be difficult to generalize its results as other ways of conducting a music intervention on a Geriatric Assessment Unit may have yielded different results. Our study was undertaken to improve the quality of care of geriatric patients hospitalized at St. Mary’s Hospital Center and to promote the *Senior-Friendly Hospital* Approach. The main goals of this approach are to prevent functional decline as well as providing tailored quality care to the older inpatients [39]. As participation in the music sessions was on a voluntary basis, the allocation to groups (exposed vs control) was not randomized. This resulted in groups with unbalanced patients’ characteristics and a selection bias. There was a difference between groups for the mean MSF score at baseline. Even if this difference was not significant, the proportion of individuals at high risk of falls based on MSF classification was higher in the exposed group compared to the non-exposed group. In addition, the change of MSF score during the hospitalisation was different between groups: it increased in non-exposed group and decreased in exposed group. This constitutes a limitation of the study. To control for this, we used the variation of MSF score ((discharge - upon admission)/ ((discharge + upon admission)/2)) × 100) as the key outcome. This solution allows us to take into consideration the difference between groups at baseline. We hope that the methodology developed for this study and its positive outcome have laid a foundation for future randomized clinical trials using music listening in the GAU setting, particularly in regard to patient’s mobility and risk of falls.

## Conclusion

We report here an association between participation in music listening sessions and a decrease in the risk of falls as measured by the MFS. One of the possible explanations for this is that music may have had a mood-enhancing effect, i.e. alleviation of depressive symptoms which have been associated with a higher risk of falls. Another possible explanation is that patients had to walk to participate in the music listening sessions, and this physical activity coupled with musical stimulation may have positively transferred to a diminished risk of falls. Further research using a randomized trial design is needed to confirm this relationship.

## Abbreviations

GAU: Geriatric Assessment Unit
MFS: Morse Fall Scale
MMSE: Mini Mental Status examination
SD: Standard deviation
